# Preparation and Characterization of Nanoparticle β-Cyclodextrin:Geraniol Inclusion Complexes

**Published:** 2018

**Authors:** Zahra Hadian, Majedeh Maleki, Khosro Abdi, Fatemeh Atyabi, Abdoreza Mohammadi, Ramin Khaksar

**Affiliations:** a *Department of Food Science and Technology, National Nutrition and Food Technology Research Institute, Shahid Beheshti University of Medical Sciences, Tehran, Iran.*; b *Department of Pharmaceutical Chemistry, Faculty of Pharmacy, Tehran University of Medical Sciences, Tehran, Iran. *; c *Nanotechnology Research Centre, Faculty of Pharmacy, Tehran University of Medical Sciences, Tehran, Iran. *; d *Research and development Department, Clear Labs Inc, Menlo Park, CA 94025, USA.*

**Keywords:** Geraniol, β-Cyclodextrin, Nanoparticle, Physicochemical, Characterization

## Abstract

The aim of the present study was to formulate β-cyclodextrin (β-CD) nanoparticles loaded with geraniol (GR) essential oil (EO) with appropriate physicochemical properties. Complexation of GR with β-CD was optimized by evaluation of four formulations, using the co-precipitation method, and the encapsulation efficiency (EE), loading, size, particle size distribution (PDI) and zeta potential were investigated. Further characterization was performed with nuclear magnetic resonance spectroscopy (^1^H NMR), differential scanning calorimetry (DSC), scanning electron microscopy (SEM) and infra-red (IR) spectroscopy analysis. Results showed that the physicochemical properties of the nanoparticles were affected by GR content in formulations that yielded nanoscale-size particles ranging from 111 to 258 nm. The highest encapsulation efficiency (79.4 ± 5.4%) was obtained when the molar ratio of EO to β-CD was 0.44: 0.13 with negative zeta potential (-21.1 ± 0.5 mV). The ^1^H-NMR spectrum confirmed the formation structure of the EO and β-CD nanoparticle complex. Complexation with geraniol resulted in changes of IR profile, NMR chemical shifts, DSC properties, and SEM of β-cyclodextrin. Inclusion complex of essential oil with β-cyclodextrin was considered as promising bioactive materials for designing functional food.

## Introduction

Geraniol is a secondary metabolite of medicinal plants that is naturally found in rose, palmarose and geranium oils. Geraniol (3, 7-dimethylocta-2, 6-dien-1-ol) is an aliphatic monoterpene alcohol which is insoluble in water. Different studies have shown that geraniol has antioxidant activity, anti-inflammatory, antimicrobial, antifungal, antiviral and apoptosis effects (1, 2). The US Food and Drug Administration confirmed it as a food additive for flavoring foods such as beverages, candies and ice creams. Flavors affect the consumption of foods, and are crucial in determining consumers’ satisfaction. Most natural and artificial flavors are volatile oils or liquids; thus, during production and storage processes their quality and quantity often change (3).

Studies indicate that geraniol oil is unstable during storage, and its autoxidation upon air exposure could form a mixture of oxidation products that produce a potent off-flavor. This phenomenon can be mitigated by encapsulation, such as the formation of inclusion complexes with cyclodextrins (CDs) (4, 5). Cyclodextrins have many advantages such as improving solubility, stability and bioavailability of drugs (6).

Molecular encapsulation of volatile or unstable food additives by using cyclodextrins has been shown to extend product shelf-life by improving these additives’ stability. For instance, their hydrophobic cavities can encapsulate organic and inorganic molecules with smaller molecular size to form various inclusion compounds in liquid- or solid-state forms, while their hydrophilic shells can generate non-inclusion complexes with larger molecular guests (7-9). Among the natural CDs, β-CD is mostly used because of its suitable cavity size for common guests with 200-800 g/mol molecular weight; it is also inexpensive and readily available (10).

During production and storage of foods, evaporation and exposure to oxygen often diminish flavors, since most natural and artificial flavors are volatile oils or liquids. CD-flavor inclusion complexes offer great potential for retaining flavor materials in a multicomponent food system. Reports have indicated that the loading capacities of β-CD-flavor complexes vary from 6% to 15% (3, 11).

There are several methods for producing an inclusion complex between CDs and bioactive compounds; the choice of a particular method depends on the guest-molecule properties, such as its availability and cost (12, 13). Neutralization, slurry, solution, co-precipitation, kneading and grinding methods are the most common techniques used for the complexation process (14). 

β-Cyclodextrin is a useful material for geraniol inclusion, due to its low bio-toxicity and high biocompatibility, solubility and bioavailability of poorly soluble compounds in oral drug delivery. Researchers have confirmed the use of CDs for protection and stabilization of different essential oils against heat, oxygen and evaporation (15-19). CDs as molecular encapsulates can also improve the flavor quality and provide a longer period of preservation than other encapsulate procedures (20).

The aim of the present study was to develop a β-cyclodextrin-based nanocarrier system for geraniol. The size, PDI, zeta potential and encapsulation efficiency of four formulations were characterized. The developed nanoparticles were evaluated by differential scanning calorimetry, infra-red spectroscopy, scanning electron microscopy, and nuclear magnetic resonance.

## Experimental

*Materials*

Geraniol (98% purity) and β-cyclodextrin (≥ 97% purity) were obtained from Sigma Company (St. Louis, MO, USA). Menthol and tetramethylsilane (TMS) were used as internal standards and purchased from Aldrich (Steinheim, Germany). Anhydrous sodium hydroxide, hydrochloric acid and dimethyl sulfoxide-d6 (DMSO-d6, purity at least 99.5%) were purchased from Merck (Merck, Darmstadt, Germany). Demineralized water (pH = 7.6 ± 0.2) was used in the study. All other solvents were of analytical reagent grade.

*Preparation of geraniol: β-cyclodextrins inclusion complexes*

The complexes of geraniol: β-cyclodextrin were prepared by precipitation according to the procedures described by Rachmawati *et al*., with minor modifications (21). Four formulations (F1 to F4) were prepared as listed in Table 1. Briefly, approximately 500 mg (0.44 mmol) of β-CD was dissolved in 20 mL of ethanol and distilled water (20: 80 v/v) at 65 °C for 30 min. Then geraniol (0.13 mmol) was added to the hydro-alcoholic solution of β-CD with continuous agitation 

(pH = 7.0 ± 0.2). Next the mixture was stirred at 300 rpm for 1 hour at 37 ºC and let stand for 4 h at room temperature. Then the mixture was sonicated for 10 minutes at 4 ºC to decrease the particle size and incubated for 12 h at 4 °C. After that time, the solution was centrifuged at 3000 rpm for 15 min to precipitate the free geraniol, the supernatant was passed through a 0.2 μm filter (Sartorius, cellulose acetate, 47 mm). The sample (F1) was placed under vacuum at 40 °C for 6 h, weighed, sealed and stored at 4 °C until physicochemical characterization. All the experiments were accomplished in triplicate and mean ± SD was reported.

*Total oil extraction*

The total amount of oil in β-CD: geraniol complex powder was measured according to Bhandari *et al*., with minor modifications (22). Briefly, 0.20 g β-CD: geraniol complex powder was dissolved in distilled water (8 mL) and hexane (4 mL) in a sealed 20 mL screw glass vial. The solution was stirred with a magnetic stirrer at 500 rpm for 20 min at 65 °C. The supernatant containing the organic phase and oil was decanted and extracted with hexane at 65 °C for several iterations. The extracted part was concentrated under nitrogen gas to approximately 1 mL and stored at 0 °C for GC analysis.

*Surface-oil extraction*

For determination of geraniol present on the surface of β-CD: geraniol complex powder, the product (1 g) was dissolved in the hexane (20 mL), as previously described by Bhandari *et al*. (16) The vial was shaken, and then stirred vigorously for 20 min. After filtering the residue was further washed with hexane (10 mL). The solvent was removed under vacuum, and the extracted oil was concentrated by nitrogen stream to approximately 1 mL and stored at 0 °C until further analysis by GC/FID.

*GC analysis*

The concentration of geraniol in the sample was determined by a Hewlett-Packard model 6890 gas chromatograph (Agilent, Wallborn*, *Germany) equipped with an FID detector and stationary phase, using HP-5 column (30 m length × 0.25 mm ID × 0.25 µm film thickness). 

Nitrogen was the carrier gas at 1 mL/min. Temperature programming was performed from 75 to 250 ºC, at 75 °C for 2 min, 75 to 110 °C at 10 °C/min and 110 to 265 °C at 40 °C/min (23).

The concentration of geraniol was quantified using standard calibration curves. Calibration curves were constructed for each compound using six different concentration levels of geraniol and the internal standard in hexane in a range of 0.5-10 μg mL^-1^.

*Entrapment efficiency and loading*

The EE and DL of the β-CD inclusion complexes for geranial were determined using the following equations according to Tao *et al.* (2).

% EE= A (mg) / B (mg) ×100                              (1)

% DL= A (mg) / C (mg) ×100                               (2)

where A, B and C are the amount of trapped geraniol (the difference between total oil and surface oil) in the complexes, the primary feeding amount of geraniol and the amount of particles was produced respectively.

*Particle size, polydispersity index and zeta potential*

β-cyclodextrin complex formulations were determined by laser light scattering (Zetasizer ZS, Malvern, UK), after suspending 5 mg of the nanoparticles in 20 mL of deionized water (21).

*Scanning electron microscopy*

A Hitachi SU3500 scanning electron microscope (Hitachi, High-Technologies Corporation, Japan) was used for surface morphology determination. The samples were attached to an aluminum stub using double-sided carbon tape and then made electrically conductive by coating them in a vacuum with thin layer of gold (33 Aº thickness), then analyzed by SEM.

*IR spectroscopy*

Geraniol, β-CD, physical mixture and β-CD: geraniol inclusion complex were scanned in the infrared spectra covering the range of 4,000-400 cm^-1^ (Spectrum one, Perkin Elmer, Waltham, MA, USA). The spectra were an average of 32 scans at a resolution of 4 cm^-1^. IR spectral analysis was performed using the KBr pellet press method, and infrared spectra were recorded on a Nicolet’s Magna-IR system 550 equipped with Nicolet’s OMNIC software. The detector was purged carefully with clean, dry helium gas to increase the signal level and reduce moisture (24).

*Differential scanning calorimetry*

Thermal analysis was performed for β-CD, geraniol, their physical mixture and the inclusion complex using a Mettler Toledo DSC system (DSC, 823E, Mettler Toledo, GM BH, Switzerland). Mettler Stare software, version 9.x, was used for data acquisition, and indium was used to calibrate the instrument. The samples (2 mg) were sealed in the aluminum pans and 

heated at the rate of 10 °C/min from -20 to 300 °C under an 8 kPa nitrogen atmosphere (25). An empty pan sealed in the same way was used as a reference. Each test was repeated three times.

*Proton nuclear magnetic resonance spectroscopy*

^1^H-NMR spectroscopy in solution is an effective method for studying geraniol: β-CD inclusion complexes. All ^1^H-NMR spectra were recorded on a Bruker at 20 °C by using DMSO-*d*_6_ (Bruker Avance AQS-300 MHz, Rheinstetten, Germany). 

The samples were located in NMR tubes 8 inches in length and 5 mm in external diameter. Geraniol (20 mg) and β-CD (20 mg) were added to 1 ml of DMSO-*d*_6_. TMS was the internal standard (δ 0.0). The mixtures were vigorously shaken at 200 rpm. 

The samples were filtered and collected as β-CD: geraniol complexes for ^1^H-NMR analysis at 25 °C (26). Spectra were recorded with 128 transients at a temperature of 25 ºC and a spectral width of 7812 Hz, a relaxation delay of 2 s and an acquisition time of 2.095 s. Data were processed using the software XWIN NMR, version 3.1*.*

*Statistical analysis*

All results are reported and displayed as mean ± standard deviation. To assess the impact of the formulation variables on the results, statistical analysis was performed using one-way analysis of variance (ANOVA) and Duncan’s multiple range tests. Differences were considered significant at *p* < 0.05. 

## Results and Discussion

*Encapsulation efficiency and loading of GR/β-CD Inclusion Complex*

**Figure 1:**
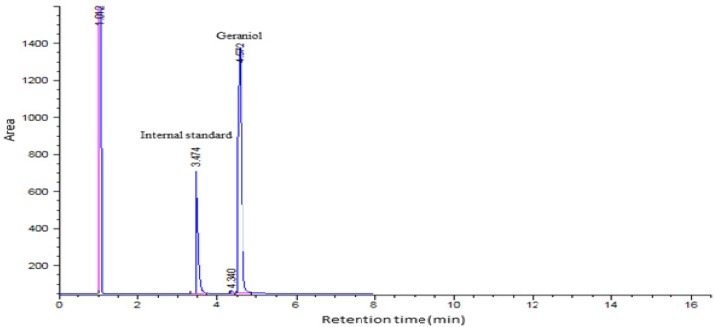
GC-FID chromatogram for geraniol analysis

The drug entrapment efficiency of β-CD: geraniol formulations were investigated by using different geraniol to β-CD ratios. Both total oil and surface oil was detected by GC/FID. The retention times of the internal standard (IS) and geraniol as illustrated in Figure. 1 were 3.47 and 4.57 min respectively. The entrapment efficiencies of geraniol in β-cyclodextrin formulations prepared with co-precipitation method are indicated in Table 1. The entrapment efficiency of the inclusion complex of β-CD: geraniol ranged from 21.6% to 79.4%. As shown in Table 1, increasing the ratio of geraniol slightly decreased encapsulation efficiency and loading of nanoparticles. The F1 formulation showed good entrapment and loading of geraniol in β-cyclodextrin. It seems that some of the reduction in the EE could be due to the evaporation of geraniol during the process; similar result has also been reported by Tao *et al*. (2).

The molar ratio will range of 1:1 to 1:10 for CD: guest. It will depend on the size and loading of the guest molecule. The results indicated that adding more geraniol did not cause more encapsulation. Statistical comparison indicated that there was significant difference (*P *≤0.05) between 0.44:0.13 and 0.44:1 ratios. Efficiency of encapsulation depends on the different amount of geraniol used similar other reports (16, 21). The amount of surface oil volatiles as determined by washing the powder with hexane ranged from 1.62 to 10.2 mg of dried powder. As indicated in Table 1, statistically significant differences were observed between three formulations in surface oil with the exception of F3 Formulation, these results are in agreement with the observations of previous studies (16, 22), that reported for the surface oil content of lemon oil and orange oil β-cyclodextrin complexes. These finding may be due to the method of extraction and the solvent used for extraction. Mortzinos *et al*., found that the EE of geraniol (0.3 mmol) encapsulated in β-cyclodextrin and in the presence of a surfactant was about 60%, which was consistent with our results (18). In contrast, they found that for the same value of thymol encapsulated within β-cyclodextrin, the EE increased to 90%; this difference may be related to β-cyclodextrin’s structure, which contains a cavity suitable for phenyl-ringed molecules such as thymol (27). 

Lopes *et al*. prepared four different values of β-cyclodextrin and a constant value of geraniol with ratios of 1.33: 1, 3.33: 1, 4.66: 1, and 6.66: 1(28). The highest EE obtained for the 6.66: 1 ratio (for 10 g β-cyclodextrin and 1.5 g geraniol) was about 34.2%.

**Table 1:** Effect of geraniol concentration on encapsulation efficiency and loading of β-CD inclusion complexes (n = 3).

**GR/β-CD** **(mole ratios)**	**Loading ** **(%)**	**EE (%)**	**Surface oil** **(mg)**	**Total oil** **(mg)**	**Recovered powder** **(mg)**
0.44: 0.13 (F1)	7.8 ± 0.70^a^	79.4 ± 5.4^a^	1.62 ±0.2^a^	17.5 ± 1.1^a^	201.2 ± 1.5^a^
0.44: 0.2 (F2)	6.9 ± 0.1^ab^	58.4 ± 0.9^b^	3.1 ± 0.3^b^	17.7 ± 1.7^a^	211.8 ± 2.2^b^
0.44: 0.4 (F3)	6.6 ± 0.28^b^	46.6 ± 2.5^c^	3.8 ± 0.4^b^	26.3 ± 1.5^b^	352.5 ± 4.1^c^
0.44 :1 (F4)	6.5 ± 0.6^b^	21.8 ± 1.9^d^	10.2 ±1.8^c^	43.0 ± 2.5^c^	502.4 ± 5.5^d^

Different superscripts within a column indicate significant differences (*P* < 0.05). Results are the average and standard deviations (SD) from three individual experiments.

**Table 2:** PDI, particle size and zeta potential of Geraniol: β-CD inclusion complexes (n = 3).

**Inclusion complexes**	**Zeta potential (mV)**	**Particle size (nm)**	**Polydispersity index**
F1	-21.1 ± 0.49^a^	111± 0.11^d^	0.16± 0.05^a^
F2	-21.6 ± 1.13^a^	165 ± 4.25^b^	0.46 ± 0.14^a^
F3	-19.4 ± 2.05^a^	173 ± 5.65^b^	0.46 ± 0.07^a^
F4	-17.4 ± 4.8^a^	258 ± 7.7^a^	0.47 ± 0.07^a^

Different superscripts within a column indicate significant differences (*P* < 0.05)

Results are the average and standard deviations (SD) from three individual experiments.

**Figure. 2.**
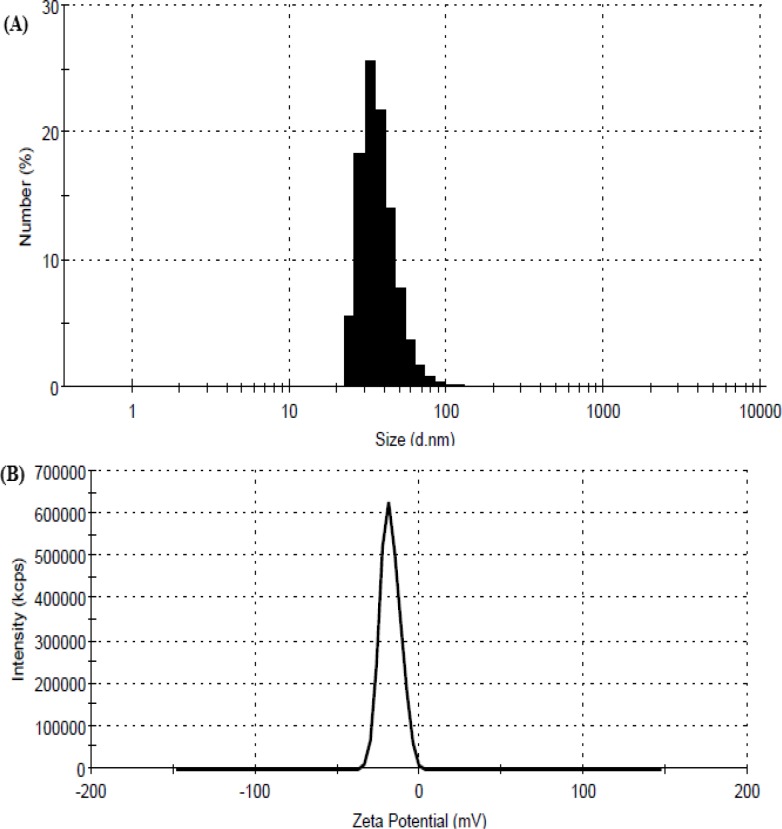
Particle size (a) and zeta potential (b) of the β-cyclodextrin:geraniol inclusion complexes (F1).

**Figure 3:**
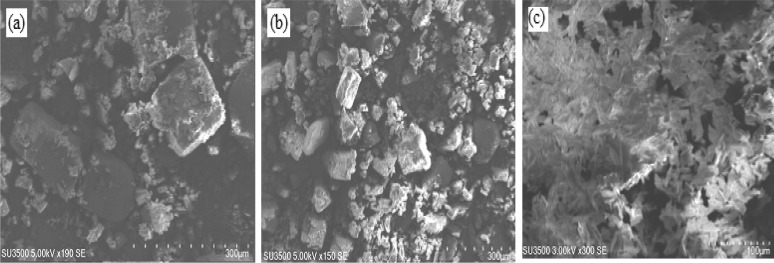
Scanning electron micrographs images of β-CD (a), β-CD:geraniol physical mixture (b) and β-CD:geraniol inclusion complexes (c

**Figure. 4:**
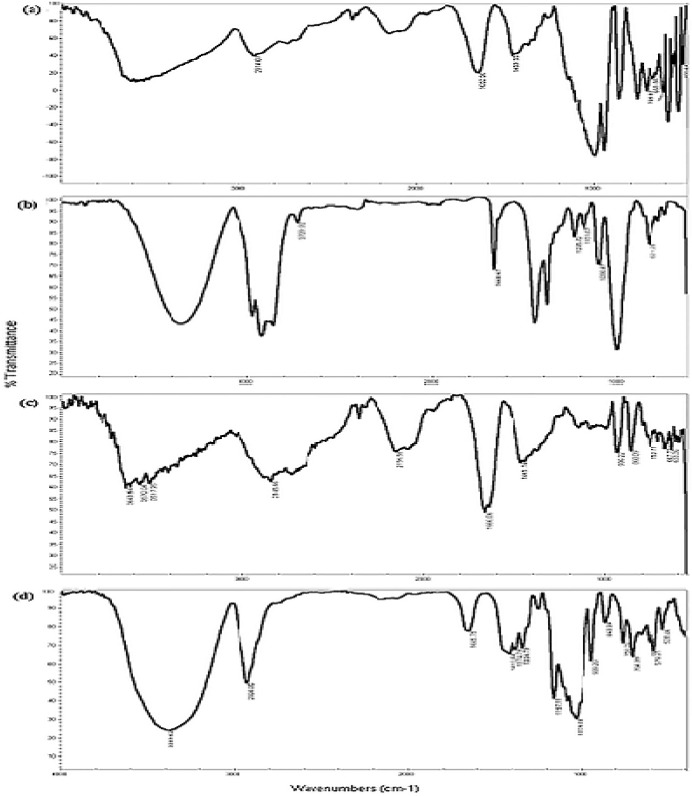
IR spectra of (a) β-CD, (b) geraniol, (c) β-CD/ geraniol physical mixture and (d) β-CD/ geraniol inclusion complex

**Figure 5:**
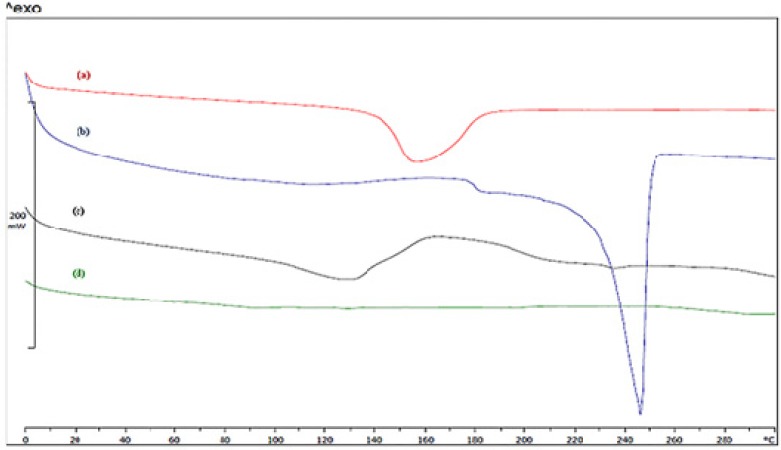
DSC thermograms of (a) β-CD, (b) geraniol, (c) β-CD/ geraniol physical mixture and (d) β-CD/ geraniol inclusion complex

**Figure 6:**
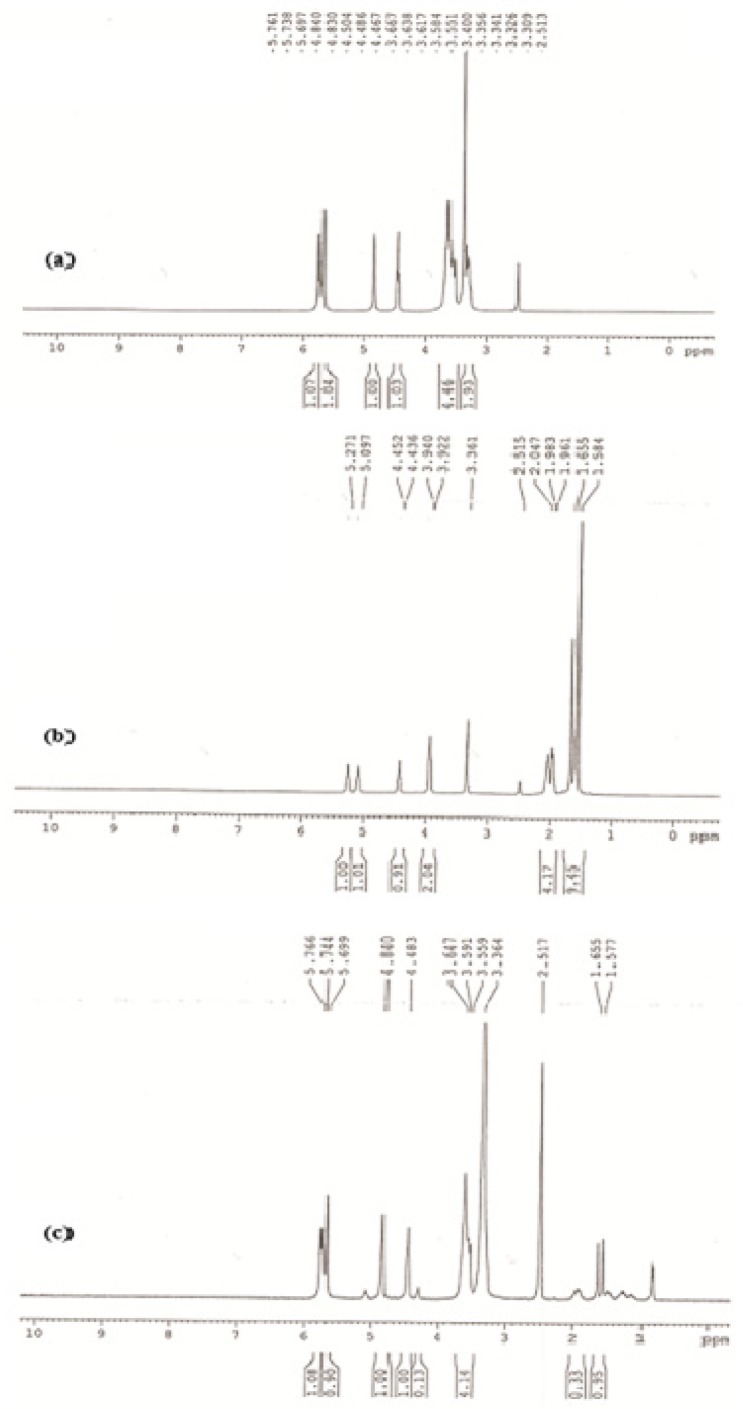
Proton NMR of spectrum of (a) β-CD, (b) geraniol, (c) β-CD/ geraniol inclusion in DMSO

Bhandari *et al*. (1998) showed that maximum encapsulation efficiency was attained when the ratio of lemon oil to β-cyclodextrin was 88: 12 (22). Our finding was consistent with other studies (21, 29). This means that by enhancing the amount of geraniol in a formulation, the value of oil remaining on the surface of β-cyclodextrin was increased and the EE % was decreased. This result may be related to the saturation capacity of the polymer matrix for guest. Reports have revealed that the inclusion stability mainly depends on geometric complementarity between encapsulated molecule and CD’s cavity. Physicochemical properties of both CD (cavity diameter, derivative nature) and guest (geometry, volume, hydrophobicity) play a crucial role in the formation of inclusion complexes. The preparation method also influenced the encapsulation efficiency. A higher efficiency was achieved for molecules that have higher binding constant with β-CD. This kind of complexation involves the drying of solid complexes after their preparation, for which different drying methods can be used. Encapsulation and drying methods are decisive in the final structure of the solid complexes. Recently, more focus has been shifted to the ICs with biodegradable polymers or copolymers because of their potential applications as novel biomaterials such as polyethylene glycol, Poly vinyl alcohol, polymer blends, and composites of organic and inorganic materials.2-Hydroxypropyl-β-cyclodextrin (HPβCD), a chemically modified β-cyclodextrin, has a greater aqueous solubility compared with its parent compound, β-cyclodextrin, and is a potential ingredient in pharmaceutical dosage forms. The possible advantages of including HPβCD in a dosage form include improved chemical stability, increased aqueous solubility and enhanced bioavailability of the guest (4, 5, 8, 30, 31).

*Particle size, PDI* *and zeta potential*

The particle size, PDI and zeta potential of four formulations containing geraniol are reported in Table 2. The nanoparticle (NP) size of four formulations (F1, F2, F3 and F4) was in the range of 111 to 258 nm and the prepared nanoparticles were uniform and monodispersed. The mean size of F1 formula was smaller than other formulations (Figure 2a).

The size of the nanoparticles (NPs) was also dependent on GR concentration. These results illustrate a significant correlation between NP EE and size; in other words, reducing the particle size increases the EE. Hashemiravan *et al*. reported an opposite relationship between EE and size of nanoparticles of β-cyclodextrin containing inulin; this result is identical to results of our study (32). Their findings indicate that lower β-CD concentration is related to higher encapsulation efficiency; thus, it may be proposed that β-CD has an optimum capacity for interaction with GR molecules. Similar observations have been reported by other researchers working on geraniol and β-cyclodextrin (18, 28). Alternatively, it has been shown that increases in β-CD concentrations induce aggregation and an increase in particle size (30, 33). In the preparation method one of the main variables that may significantly affect the interaction between β-CD and hydrophobic molecules has been examined by other researchers working on essential oil and β-CD (34). 

In our study, the PDI of four formulations did not show a significant difference (*p *> 0.05). The polydispersity index (PDI) value of all formulas ranging from 0.16 - 0.47 that represented the F1 is stable formulation and having homogeneity of particles. These results indicated that geranial concentration can markedly influence on particle size and PDI characteristics of the inclusion complexes. Agglomeration as a consequence of the self-assembly of cyclodextrin complexes in water, is one of the reason of significant increase in mean size and PDI of formulation (35). 

Zeta potential is another major characteristic of nanoparticles that may have affect their stability. The zeta potential of the inclusion complex in this study was negative due to the presence of geraniol. As illustrated in Table 2, the values of negative zeta potential on the nanoparticles ranged from -19 to -21 mV. These β-CD–geraniol nanoparticles have a negative surface charge (Table 2) indicating by the zeta potential value. Negative charge at the surface shows that the molecular aligning of the amphiphilic β-CDs are such that the unplaced –OH groups are directing towards the aqueous surrounding rendering a potential surface hydrophilicity (21). The zeta potential value for the F1 formulation was more negative (Figure 2b).

The zeta potential of the nanoparticles decreased as the geraniol concentration decreased from a guest-to- β-CD ratio of 0.44:0.13 to 0.44:1 (mmol/mmol). 

The zeta-potential values of the four formulations were not significantly different (*p *> 0.05). This reduced negative charge may be attributed to the low entrapped guest molecules with higher β-cyclodextrin concentrations. Zeta potential is an indicator of the charge presence on the surface of the nanoparticle, thereby indicating the degree of stability. Dispersion system stability is possible when the zeta potential value is near −30 or + 30mV (36).

*Scanning electron microscopy*

The SEM micrographs of pure β-CD, β-CD/GR physical mixture and β-CD/GR inclusion complexes are shown in Figure 3. Pure β-CD shows as crystalline particles with different sizes without a characteristic shape (Figure 3a). The particle shapes and morphologies of the corresponding physical mixture (Figure 3b) were similar to pure β-CD. As illustrated in Figure 3c, the geraniol and β-cyclodextrin inclusion complex has great uniformity in its crystal and regularity in its shapes that were also confirmed by the RI and NMR analysis, similar results were reported by Menezesa *et al*. (15).

*Infrared spectroscopy*

The variation of the shape, shift or intensity of IR absorption peaks of the guest or host can produce information about the occurrence of inclusion-complex formation. The IR spectra for geraniol (Figure 4a) showed absorption bands at 3354 cm^-1^ (O-H stretching vibration), 2968 cm^-1^, 2919 cm^-1^, 2729 cm^-1^ (C-H stretching vibration), 1668 cm^-1^, 1444 cm^-1^ (C = C stretching vibration), 1235 cm^-1^ (C-C stretching vibration) and 1118 cm^-1^, 1098 cm^-1^ and 1001 cm^-1^ (C-O stretching vibration). 

The IR spectra for β-cyclodextrin (Figure 2b) indicated absorption bands at 3582 cm^-1^ (O-H stretching vibration), 2914 cm^-1^ (C-H stretching vibration), 1652 cm^-1 ^(H-O-H stretching vibration), 1159 cm^-1^ (C-O stretching vibration) and 1091 cm^-1 ^(C-O-C stretching vibration). As shown in Figure 4c, changes in the shape, position and intensity of the absorption bands of pure β-CD, the physical mixture and the inclusion complex were detected. The physical mixture (Figure 4c) presented a combination of the individual patterns of β-CD and GR, although there were obvious changes in the spectrum of the inclusion complex of geraniol and β-cyclodextrin (Figure 4d) the absorption peaks were shifted to lower frequencies at 3389 cm^-1^, 2924 cm^-1^, 1645 cm^-1^ and 1416 cm^-1^. These changes can indicate the formation of intramolecular hydrogen bonds in the β-CD: GR inclusion complex (37). The disappearance or reduction of the absorption intensities of the corresponding bands suggested that the functional groups of the drug were included within the apolar cavity of β-CD in the complex. The findings were consistent with the reports of Menezes *et al*. and Wang *et al*. (15, 37)

*Differential scanning calorimeter*

DSC studies were performed to assess the physical state of the geraniol and β-cyclodextrin inclusion complex, their physical mixture, pure geraniol and pure β-cyclodextrin.DSC thermograms of samples are presented in Figure 5. The guest substance may be changed by melting, evaporation, decomposition, oxidation, or polymorphic transition (38, 39).

In general, complexation results in the disappearance of endothermic peaks, appearance of new peaks, and peak broadening or shifting to different temperatures, which indicate a change in the crystal-lattice, melting, boiling or sublimation points. DSC thermograms of pure β-cyclodextrin (Figure 5a) and geraniol (Figure 5b) showed an endothermic melting peak at 248 °C and 155 °C; these results may be related to oxidation or the elimination of water (40). The thermogram of the physical mixture of β-CD and GR (Figure 5c) shows two endothermic peaks at about 130 °C and 240 °C, which was similar to that of pure β-CD and geraniol, this may indicate less or no interaction between the drug and β-CD in the physical mixture. In contrast, the DSC curve for the formulation F1 (Figure 5d) did not show any endothermic peak, which suggests that the guest incorporated into the nanoparticles and confirms the formation of a host-guest inclusion complex. Elimination of the free-geraniol and β-cyclodextrin peak, indicating the formation of a complex between these two compounds, has been reported by Jin and Wang *et al*. (8, 37). 

DSC is a preferred method to prove the formation of an inclusion complex of β-cyclodextrin and a guest molecule. If the guest molecule is well placed in a polymer matrix, no exothermic or endothermic peak should be observed in the guest-polymer or bioactive-polymer thermogram. Similar observations have been reported when formed inclusion complexes with β-CD there is no endothermic peak or melting peak in the thermogram (37, 40).

^1^ *H NMR analysis*

The structural characterization of GR/β-CD complex sample at 1: 5 molar ratios was studied by using ^1^H NMR. Initially, the characteristic peaks correspond to protons of pure β-CD and geraniol, were determined from their ^1^H NMR spectra. Insertion of a guest molecule into the hydrophobic cavity of β-CD cavity in the a polar region results in the modification of ^1^H NMR frequencies. Major changes in the chemical-shift values of the inner β-CD protons more specifically, H3 and H5 located inside the cavity, or H6 on the cavity rim indicate the formation of an inclusion complex, but the chemical-shift values of the outside protons (H1, H2, and H4) are relatively unaffected (41). This should occur in chemical-shift changes of the inside protons. 

The ^1^H NMR spectrum of β-CD in the absence and presence of GR is shown in Figures. 6a, b and c. All ^1^H NMR spectra characteristic peaks of geraniol and β-CD confirmed the complex of geraniol and β-CD sample. The inclusion complex is formed by hydrophobic forces, Vander Waals interaction, the release of ring strain, and modification in solvent surface, tensions and hydrogen bonds, which lead to stability of the inclusion complex (42, 43). As indicated in Figure. 4c, the upfield in signal of the H3 and H5 of β*-*CD was observed on the inner side of the cavity; this indicates the penetration of geraniol molecules inside the cavities. 

The β-CD resonance occurs between 3.2 and 4.8 ppm. After complexation of geraniol molecules with β-CD, the chemical shifts of neither the guest nor the host indicated a significant change. The effects of β-CD on the 1H chemical shifts of GR indicate a slight shift in the resonance of H-1, H-2, H-3, H-4, H-5 and H-6 protons of β-CD. These results could suggest the presence of one geraniol molecule inside the β-CD cavity.

## Conclusion

Nanoparticle (NP) complexes of geraniol and β-cyclodextrin were successfully prepared using co-precipitation. The encapsulation efficiency of the NP complexes was optimized at different ratios of GR to β-CD. The data demonstrate that encapsulation efficiencies of 79.75% and 21.81% were obtained at ratios of GR to β-CD of 0.44: 0.13 (mmol/mmol), respectively. We further observed that the size of the NP complexes and the encapsulation efficiency of the guest increased concurrently with increasing ratios of GR to β-CD. The results of DSC, SEM and IR, along with large variation of chemical shifts from protons located around the interior of the hydrophobic cavity from ^1^H NMR, provided clear evidence of inclusion complexation of GR and β-CD nanoparticles. 
